# Curing-state-dependent rheology governs electrospinning of silicone elastomers for hydrophobic surface coatings

**DOI:** 10.1007/s10856-026-07125-z

**Published:** 2026-11-05

**Authors:** Jan F. Drexler, Yan Zhang, Florian Rummel, Gesine Hentschel, Tom Bode, Marc Mueller, Gerrit Paasche, Birgit Glasmacher

**Affiliations:** 1 Institute for Multiphase Processes, Leibniz University Hannover, An der Universität 1, Garbsen, 30823 Germany; 2 NETZSCH-Gerätebau GmbH, Wittelsbacherstraße 42, Selb, 95100 Germany; 3 Department of Otorhinolaryngology, Head and Neck Surgery, Hannover Medical School, Carl-Neuberg-Straße 1, Hannover, 30625 Germany; 4 Lower Saxony Center for Biomedical Engineering, Implant Research and Development, Stadtfelddamm 34, Hannover, 30625 Germany; 5 Hearing4all, Hannover Medical School, Carl-Neuberg-Straße 1, Hannover, 30625 Germany

## Abstract

Fibrosis around cochlear implant electrode arrays increases impedance and disrupts predictable stimulus propagation. Electrospun hydrophobic silicone coatings represent a promising strategy to mitigate fibrosis. However, electrospinning silicone elastomers remains challenging because their low glass transition temperature causes deposited fibers to collapse before curing is complete. Previous approaches have focused on developing in situ curing systems, such as heated collectors. Although fiber-like structures have been generated, nonwovens with interconnected pores have not yet been achieved. A pre-curing-based electrospinning strategy was developed that enables the fabrication of pure silicone elastomer nonwovens without the need for polymer blends, sacrificial carrier polymers, or coaxial spinnerets. Sylgard 184 dilutions in acetone and tetrahydrofuran were electrospun at different pre-curing times prior to gelation and characterized rheologically to investigate the influence of curing-induced rheological evolution on electrospinnability. Initially, the dilutions exhibited near-Newtonian flow behavior. Shortly before loss of flowability, the maximum first normal stress difference increased to 3228 Pa for acetone- and 6722 Pa for tetrahydrofuran-based systems, accompanied by increased storage modulus and more pronounced shear-thinning behavior. This rheological transition coincided with the transition from electrospraying to electrospinning. By controlling the curing time, nonwoven structures with well-defined fiber geometry and interconnected pores were successfully fabricated from Sylgard/tetrahydrofuran systems. Fiber diameters ranging from 21 µm to 136 µm were achieved by adjusting the diluent and curing time. These findings demonstrate that precise control of rheological evolution during curing enables the fabrication of structurally defined electrospun silicone elastomer nonwovens, providing a foundation for anti-fibrotic coatings on cochlear implants.

**Figure:**
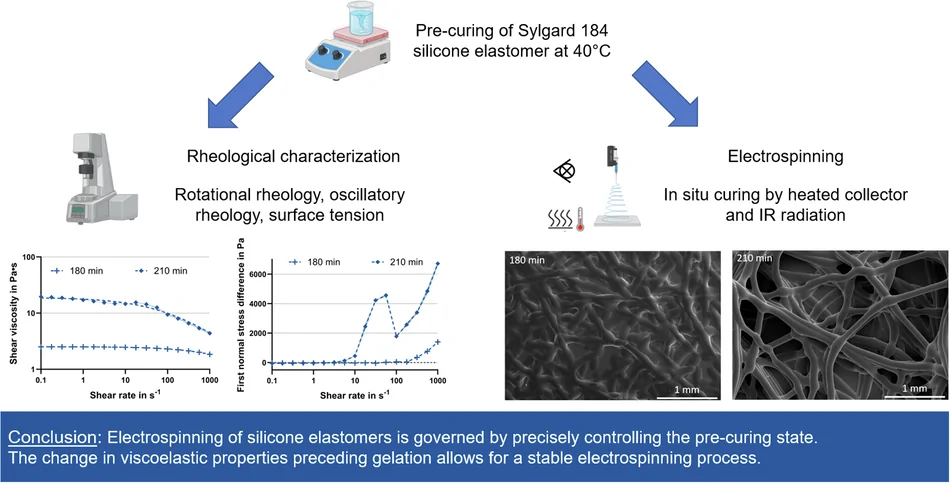

## Introduction

Cochlear implants (CIs) are used to restore functional hearing in partially or totally deafened patients. With more than one million treated patients, CIs represent the most clinically successful neuroprosthesis [1]. Following CI implantation, insertion trauma and the foreign body reaction lead to the formation of a fibrotic capsule. This results in increased impedance, unpredictable stimulus propagation and increased energy consumption [2–4]. Therefore, it is desirable to reduce connective tissue growth around the CI electrode array. The growth of fibroblasts on the materials used in the CI electrode array, platinum and poly(dimethyl siloxane) (PDMS), was investigated by Reich et al. The authors demonstrated that fibroblasts exhibited reduced growth on PDMS compared to platinum [5]. Another approach to reduce fibroblast adhesion and connective tissue growth on stimulation electrodes is surface microstructuring [5–7]. Modifying the surface of CI electrodes by addition of a structured, porous PDMS layer combines both of these approaches. Electrospun nonwovens are particularly well suited for this application. They are characterized by interconnectivity of pores and high surface-to-volume ratios. These properties may improve wetting of the nonwovens by perilymph fluid, a necessary condition for stimulation with an acceptable increase in impedance.

Electrospinning is commonly used to process thermoplastic polymers. Only a limited number of studies have reported the electrospinning of elastomers, such as PDMS. In most cases, PDMS is combined with thermoplastic polymers during the electrospinning process, either in a coaxial configuration [8–10] or as part of a blend or copolymer [11–15]. Electrospinning of pure PDMS poses significant challenges. Due to their low glass transition temperature, uncured PDMS systems are liquid at room temperature, which leads to a loss of structure in the electrospun fibers. Conversely, fully cured PDMS elastomers no longer exhibit sufficient flowability and become insoluble.

Two principal approaches have been reported for producing pure silicone fiber structures by electrospinning: (i) Using highly branched silicone resins with a glass transition temperature above room temperature [7, 16, 17]. (ii) Electrospinning of PDMS in a sol-gel process [18, 19]. Luo et al. successfully electrospun polymethylsilsesquioxane (PMSQ), a highly branched silicone resin that is solid at room temperature and soluble in various solvents [16]. Dencker et al. used a similar approach to modify the surface of CI electrodes by electrospinning of Silres MK, a methyl silicone resin. Curing was achieved by heating the fibers post-process at 200 °C for 1 h. It could be demonstrated that surface modification with hydrophobic silicone fibers reduces fibroblast growth and only slightly increases impedance by less than 120 Ω [7]. Kim et al. introduced a process for electrospinning PDMS elastomer using a sol-gel method. A prepolymer solution was prepared by mixing PDMS with tetraethyl orthosilicate in tetrahydrofuran (THF) and nitric acid. A viscous PDMS sol was produced by hydrolysis and evaporation of solvent at 80 °C. Electrospun nonwovens were cured by heat treatment after electrospinning [18].

Both approaches present challenges for their application as implant surface coatings. They require a second curing step after initial fiber fabrication. This additional curing step may lead to a further loss of fiber structure if the glass transition temperature is exceeded. After the sol-gel processes and the condensation curing reaction of the silicone resins, any by-products or residues must be sufficiently removed. Furthermore, structures produced from cured silicone resins exhibit more brittle material behavior compared to elastomers [20]. Given these limitations, using addition-curing silicone elastomers for electrospinning is preferable. These materials cure without generating by-products, exhibit minimal shrinkage, possess the elastic properties typical of silicone elastomers and are available as medical-grade products. Haerst and Seitz et al. presented an approach for electrospinning Silpuran 2430, a medical grade PDMS elastomer. The authors were able to achieve fiber-like structures by using acetone as a diluent to reduce viscosity and a heated plate collector (100 °C) to accelerate curing [21]. However, it was not possible to produce a nonwoven with interconnected pores, as the silicone spread out before complete curing could be achieved and thus filled fiber interspaces. For the application as a surface coating for CI electrodes, it is crucial to ensure pore interconnectivity of the nonwoven to enable electrical stimulation through contact of the electrodes with the perilymph fluid. Therefore, improved methods are required to produce silicone elastomer nonwovens with well-defined fiber morphologies and interconnected pore structures.

Process stability and fiber morphology in the electrospinning process strongly depend on the viscoelastic rheological properties of the fluid used. When transferring the electrospinning process from thermoplastic polymers to elastomer systems, rheological properties become a critical factor governing process stability and fiber shape retention. Because curing occurs over a finite time scale, deposited fibers can remain susceptible to deformation after reaching the collector. The viscosity and elasticity of the precursor solution determine its resistance to flow, stress relaxation and coalescence, thereby affecting the preservation of the as-spun fibrous morphology until curing is complete. Previous approaches to electrospinning silicone elastomers were limited to using the elastomer systems immediately after mixing them, at the start of the curing reaction [21, 22]. However, exploiting the change in rheological properties during the curing reaction offers the potential to modify electrospinning process stability, improving fiber morphology and structural integrity. This study investigates how the curing state of silicone elastomer dilutions, from initial homogenization to loss of flowability, affects rheological properties, electrospinnability and fiber shape retention. The study was based on the following hypotheses:

(i) Electrospinnability of silicone elastomer dilutions is governed by a late-stage curing window defined by viscoelastic thresholds.

(ii) Thresholds and morphologies within the processability window can be adjusted by selection of the diluent.

## Materials and methods

### Materials

Sylgard 184 (Dow Corning, supplied by Mavom), a two-component addition-curing PDMS elastomer, was used as the model silicone elastomer in this study. The elastomer base and curing agent were mixed in a weight ratio of 10:1 for all experiments. Acetone (Carl Roth, 99.5%) and THF (Carl Roth, 99.5%) were used as diluents without further purification. The two diluents were selected because they differ in solvent quality and electrical conductivity, allowing investigations of their influence on the rheological evolution and electrospinnability of silicone elastomer dilutions.

### Preparation of silicone elastomer dilutions

The silicone elastomer dilutions were prepared by initially mixing the curing agent with the diluent on a magnetic stirrer (MR3001, Heidolph) at 100 rpm for 10 min at room temperature. This step was performed to improve mixing of the more polar diluents with the non-polar silicone base. The polymer base was subsequently added, and the dilutions were placed in a heating cabinet (UF450TS, Memmert) on a magnetic stirrer at 40 °C to accelerate curing. During the first 30 min, the mixtures were stirred at 400 rpm to ensure complete homogenization. The stirring speed was then reduced to 100 rpm. Dilutions with concentrations of 2, 3 and 4 g/ml were investigated for both diluents (based on the total mass of polymer base and curing agent). An additional concentration of 2.5 g/ml was investigated for acetone because the 2 g/ml acetone dilution did not exhibit stable electrospinning. This additional concentration enabled comparison of three spinnable acetone formulations with the three spinnable THF formulations.

### Investigation of time-dependent rheological properties

To determine the time-dependent rheological properties of the silicone elastomer dilutions, samples were extracted at defined time points preceding gelation (see Table 1). Immediately after sampling, the specimens were stored at -24 °C to minimize further curing before characterization. Rheological measurements were performed using a Kinexus Prime Ultra+ rotational rheometer (NETZSCH) equipped with an active hood cartridge and an active solvent trap to minimize evaporation of THF and acetone during measurements. A cone-plate geometry with a cone base diameter of 40 mm and a cone angle of 1° was used. The sample volume added per measurement was 0.35 ml. After loading, the sample was equilibrated at 20 ± 0.1 °C for 2 min. The following measurements were performed:

**Table 1:** Overview of the electrospinnability and rheological properties of all the samples tested

Sample	η_0_	SI	$\dot{\gamma }$_onset_	N_1,max_	G’(10 s^-1^)	δ(10 s^-1^)	Regime	F_D_
	Pa·s		s^-1^	Pa	Pa	°		µm
A 2 400	0.67	0.18	1000	146	2.61	86.3	Spraying	
A 2 420	0.85	0.13	316.2	285	4.24	85.1	Transition	
A 2.5 260	1.72	0.39	177.8	1016	11.28	83.4	Spinning	26.05
A 2.5 280	4.45	1.38	56.4	2423	42.11	74.8	Spinning	36.73
A 3 200	2.29	0.45	316.2	1328	6.81	85.7	Spinning	21.52
A 3 210	6.21	1.56	31.6	3202	33.21	78.8	Spinning	32.39
A 4 0	0.26	0	-	-			Spraying	
A 4 160	1.93	0.19	1000	582	7.61	85.4	Spraying	
A 4 170	2.14	0.16	562.4	625	6.08	87.4	Transition	
A 4 180	3.36	0.29	316.2	1924	19.69	84.2	Spinning	35.76
A 4 190	6.02	0.73	177.8	3228	62.85	78.8	-	
T 2 230	3.5	1.24	17.8	2083	37.3	75.5	Spinning	49.1
T 2 240	80.51	31.25	0.6	5040			-	
T 3 60	0.32	0.05	-	-	3.79	84.2	Spraying	
T 3 180	1.36	0.25	1000	461	2.47	88.4	Transition	
T 3 200	5.46	1.34	56.2	2879	55.05	76.7	Spinning	65.29
T 4 0	0.08	0	-	-			Spraying	
T 4 180	2.52	0.36	316.2	1404	12.04	85.5	Transition	
T 4 190	4.31	0.69	100	2550	31.6	81.3	Spinning	66.22
T 4 200	6.89	1.3	31.6	3645	66.92	77.1	Spinning	76.22
T 4 210	18.98	3.35	10	6722	128.7	71	Spinning	135.88

Two samples (Ac 4 190 and T 2 240) lost flowability during the electrospinning process before fiber deposition could be completed. Rheological characterization was still possible because the measurements were initiated immediately after sampling, before loss of flowability occurred. Median values are displayed. Samples are named according to diluent, elastomer concentration in g/ml and curing time in minutes

(i) Shear rate tables in a shear rate range from 0.1 s^-1^ to 1000 s^-1^ with 3% deviation to the equilibrium value for 10 s as stability criterion;

(ii) Amplitude scans in oscillatory mode in a shear strain range from 0.1% to 100% at a frequency of 10 Hz;

(iii) Frequency scans in oscillatory mode in a frequency range from 10 Hz to 0.1 Hz at a shear strain of 1%.

Surface tension was determined by drop shape analysis (FM40 EasyDrop, Kruess) in the pendant drop method.

### Electrospinning of silicone elastomer dilutions

**Fig. 1:**
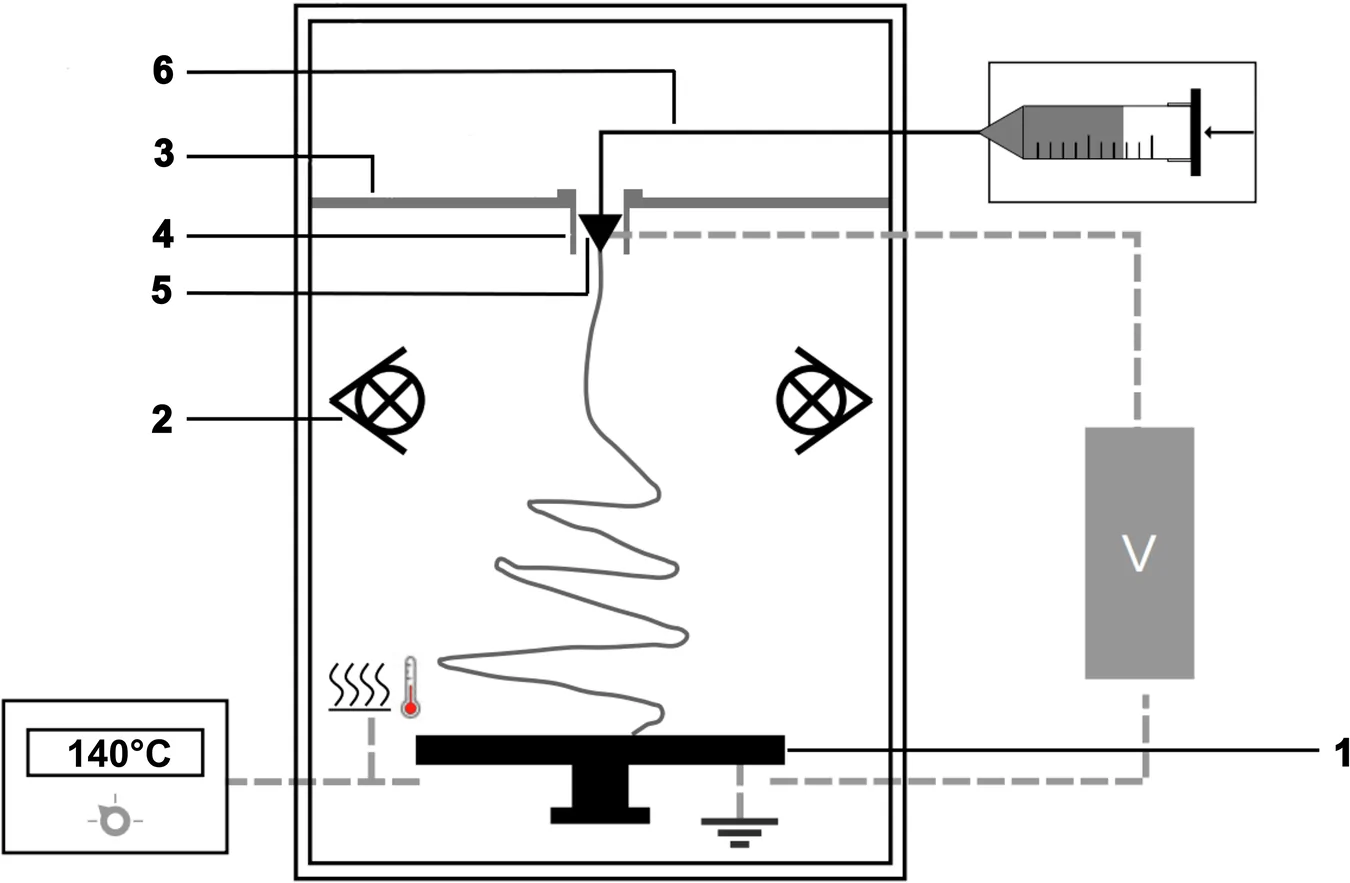
In situ curing system for electrospinning of silicone elastomer dilutions. The fibers were cured by a plate collector (1) equipped with heating foils and by two infrared lamps (2). A control unit outside the system enabled temperature regulation of the heated plate collector. A perforated plate (3) and a funnel (4) were added to avoid heating of the cannula (5) and polymer line (6)

Samples for electrospinning were collected from the curing process at defined time points (see Table 1). After cooling to room temperature, the samples were used immediately for electrospinning. Electrospinning was performed in a custom-build vertical top-down setup. The process parameters were kept constant throughout all experiments. The polymer dilution was delivered at a flow rate of 1 ml/h using a syringe pump (Fusion Touch 200, Chemyx). A voltage of -25 kV was applied to the cannula (21 G, Sterican) using a high-voltage power supply (AU-30N1-LC(220 V), Matsusada). The distance between the cannula tip and the collector was 250 mm. To preserve the fibrous morphology during deposition, accelerated curing of the silicone was required throughout the electrospinning process. For this purpose, an in situ curing unit consisting of a heated plate collector and two infrared lamps was developed. A schematic of the electrospinning setup is shown in Fig. 1.

The plate collector used was equipped with two heating foils (TRU components, supplied by Conrad). Its temperature was monitored using a K-type thermocouple (DollaTek). The heating foils and thermocouple were connected to an external controller, allowing temperature regulation up to 160 °C. Two infrared lamps (DERNORD, 150 W) were positioned at the level of the polymer jet. To prevent heating of the cannula and polymer supply line, a perforated plate and a funnel were used to shield them. All experiments were performed using a collector temperature of 140 °C under ambient laboratory conditions, with relative humidity ranging from 32% to 56% and ambient temperature ranging from 19.5 °C to 22.3 °C.

### Morphological examination

The morphology of the electrospun fibers was examined by scanning electron microscopy (SEM, S-3400N, Hitachi). Preliminarily, the fibers were sputter-coated with gold for 40 s using a sputter coater (JFC-1300, Jeol). Fiber diameters were determined by image analysis using AxioVision software (Carl Zeiss, Germany). At least 30 individual fibers were evaluated for each experimental condition.

### Analysis

The results were analyzed and plotted using GraphPad Prism (version 10.6.1). Rheological data are presented as envelopes showing the median and total range if multiple measurements could be performed with the same dilution. Shear viscosity was described using the Cross model according to Eq. (1). The Cross model provides a suitable description of fluids exhibiting at least one Newtonian plateau and a shear-thinning regime. For the uncured samples, measurements at low shear rates were affected by operation near the rheometer torque limit. Nevertheless, the Cross model adequately described the measured viscosity curves, with coefficients of determination of 0.92 for acetone-based dilutions and 0.97 for THF-based dilutions. However, because the onset of shear thinning varied considerably between samples, its application for quantifying and comparing shear thinning behavior is limited. Therefore, a shear-thinning index SI was developed to quantify this effect according to Eq. (2). The index was calculated from the zero-shear viscosity (η_0_), obtained from the Cross model fit, and the minimum measured viscosity (η_min_).

1 $${{{{\rm{\eta }}}}}\left(\dot{\gamma }\right)={{{{{\rm{\eta }}}}}}_{\infty }+\frac{{{{{{\rm{\eta }}}}}}_{0}-{{{{{\rm{\eta }}}}}}_{\infty }}{1+{({{{{\rm{\lambda }}}}}\dot{\gamma })}^{n}}$$

2 $${{{{\rm{SI}}}}}=\frac{{{{{{\rm{\eta }}}}}}_{0}-{{{{{\rm{\eta }}}}}}_{\min }}{{{{{{\rm{\eta }}}}}}_{\min }}$$

The first normal stress difference N_1_ was calculated from the measured normal force F_z_ and the cone radius R according to Eq. (3). To ensure comparability, the largest absolute value for N_1_ within the shear rate range from 0.1 s^-1^ to 1 s^-1^ was defined as the measurement noise. The onset of N_1_ was then identified as the first measured value exceeding three times this noise level.

3 $${{{{{\rm{N}}}}}}_{1}=\frac{2{F}_{z}(\dot{\gamma })}{\pi {R}^{2}}$$

## Results

### Time-dependent rheological evolution of silicone elastomer dilutions

#### Rotational rheology

**Fig. 2:**
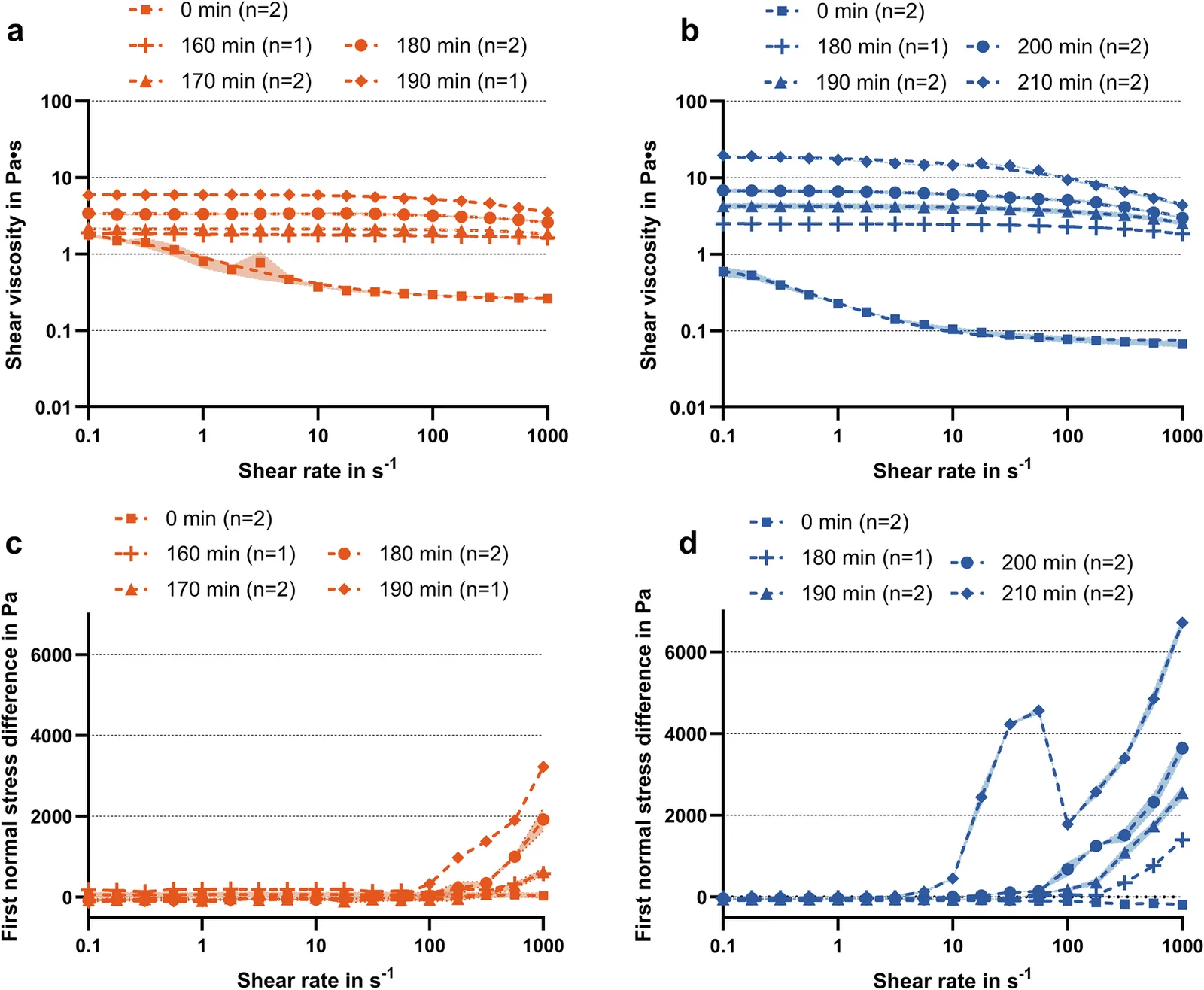
Temporal evolution of shear viscosity (**a**, **b**) and first normal stress difference (**c**, **d**) of silicone elastomer dilutions with a concentration of 4 g/ml in acetone (orange, **a**, **c**) and THF (blue, **b**, **d**). For viscosity measurements, the dashed lines indicate the fit according to the Cross model (see Eq. (1)). If several measurements could be performed with the same dilution, the data points indicate the median values and the envelope curves indicate the total range

Figure 2 shows the temporal evolution of shear viscosity and first normal stress difference for 4 g/ml silicone elastomer dilutions in acetone and THF. Results for all additional concentrations are provided in the supplementary materials (Figs. S1-S3, online resource 1). To enable quantitative comparison, Table 1 summarizes the zero-shear viscosity (η_0_), shear-thinning index (SI), onset shear rate ($\dot{\gamma }$_onset_), and maximum first normal stress difference (N_1,max_) for all formulations. The zero-shear viscosity was obtained from fits using the Cross model. All datasets with replicate measurements exhibited high reproducibility and low variability.

For both diluents, curing resulted in a continuous increase in viscosity accompanied by increasingly pronounced shear-thinning behavior. These changes accelerated markedly immediately before loss of flowability. At early curing stages, the viscosity remained nearly constant at higher shear rates, indicating near-Newtonian behavior. For shear rates below 100 s^-1^, however, equilibrium could not be reached for uncured samples due to operation near the rheometer torque limit. Additional experimental factors, such as surface tension at the edge of the sample, may also have contributed to the deviations at low shear rates.

The evolution of elasticity was reflected in the first normal stress difference. Initially, no measurable elastic response was observed within the investigated shear rate range. With increasing curing time, a characteristic threshold behavior emerged: Above a critical shear rate, the first normal stress difference increased sharply. Simultaneously, the onset shear rate shifted towards lower values and the maximum measurable first normal stress difference increased, indicating progressive development of elastic stresses during curing.

A comparison of the diluents revealed clear differences in rheological behavior. THF-based dilutions exhibited higher shear viscosity, stronger shear thinning, earlier onset of elasticity, and larger first normal stress differences compared to acetone-based systems within the investigated time range. For acetone-based dilutions, the zero-shear viscosity increased from 0.26 Pa·s immediately after preparation to 6.02 Pa·s after 190 min of curing. In THF-based dilutions, the zero-shear viscosity increased from 0.08 Pa·s to 18.98 Pa·s after 210 min. Simultaneously, the shear-thinning index increased from 0 to 0.73 for acetone-based systems and from 0 to 3.35 for THF-based systems, indicating a substantially stronger development of shear-thinning behavior in THF. Regarding the first normal stress difference, dilutions with both diluents did not exhibit a measurable elasticity immediately after preparation. Preceding the gel point, the dilution in acetone exhibited elasticity onset at a shear rate of 177.8 s^-1^ and a maximum first normal stress difference of 3228 Pa. The 4 g/ml THF dilution exhibited an elasticity onset at 10 s^-1^ and a maximum first normal stress difference of 6722 Pa. Although the loss of flowability occurred 20 min later for the THF dilution shown here, the same trend was observed for all concentrations investigated.

#### Oscillatory rheology

**Fig. 3:**
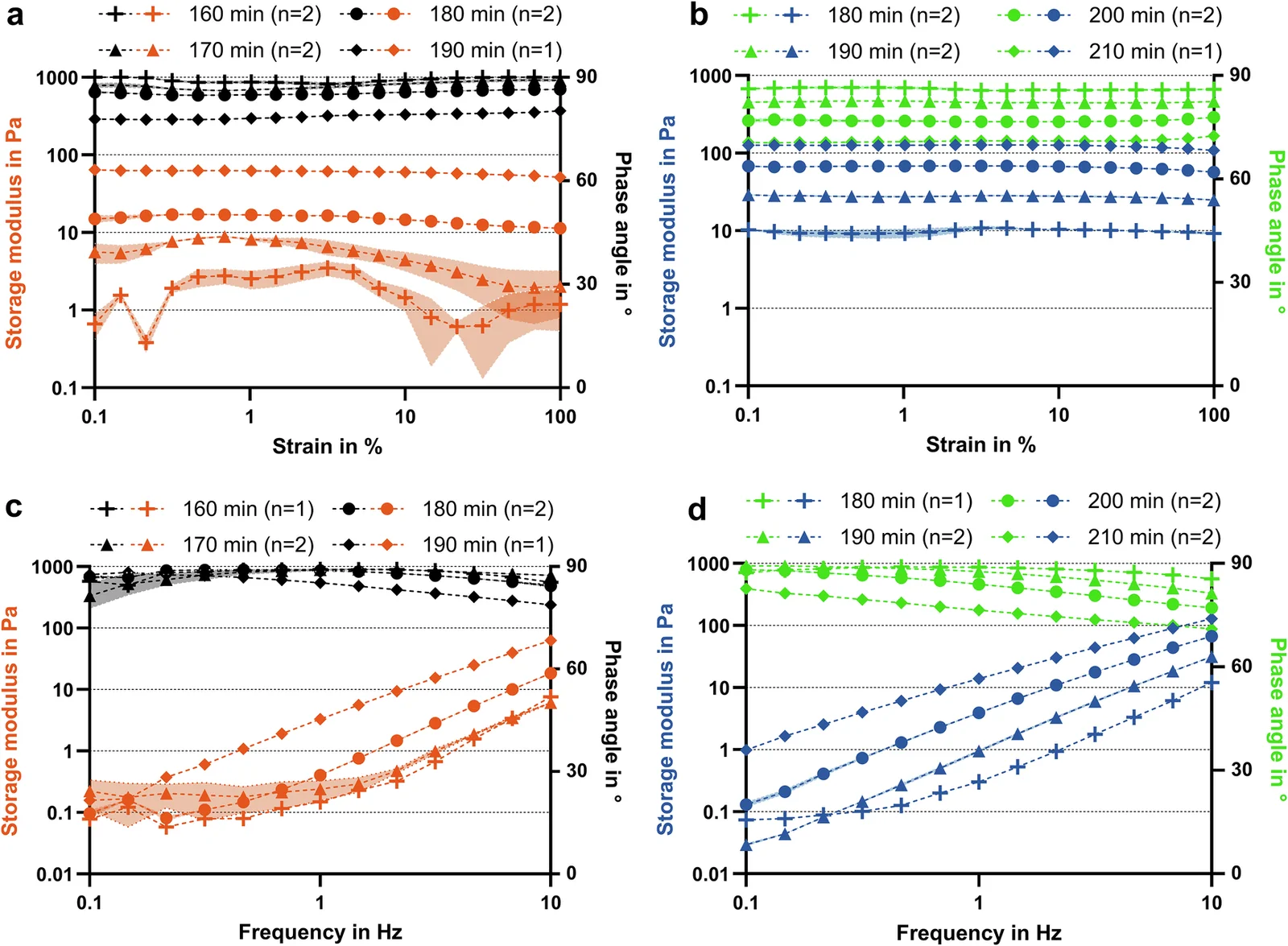
Storage modulus and phase angle of silicone elastomer dilutions with a concentration of 4 g/ml in acetone (**a**, **c**) and THF (**b**, **d**), measured during amplitude sweeps (**a**, **b**) and frequency sweeps (**c**, **d**). The storage modulus and phase angle are shown in orange and black, respectively, for acetone dilutions, and in blue and green, respectively, for THF dilutions. If several measurements could be performed with the same dilution, the data points indicate the median values and the envelope curves indicate the total range

The results of the amplitude and frequency scans for 4 g/ml silicone elastomer dilutions are shown in Fig. 3. Results for the remaining concentrations are provided in the supplementary materials (Figs. S4–S6, online resource 1). Table 1 summarizes the storage modulus (G’) and phase angle (δ) at a frequency of 10 Hz for all formulations. Across all investigated curing states, the samples exhibited predominantly viscous behavior, as indicated by phase angles exceeding 70°. Nevertheless, curing induced a gradual increase in storage modulus and a corresponding decrease in phase angle, demonstrating the progressive development of elasticity. Similar to the steady shear measurements, the most pronounced rheological changes occurred immediately before loss of fluidity.

Amplitude scans showed no characteristic decrease in storage modulus with increasing strain amplitude, indicating a broad linear viscoelastic region extending up to 100% strain. Frequency sweeps revealed increasing storage modulus and decreasing phase angle with increasing frequency. Greater fluctuations were observed for acetone dilutions at curing times of 160 and 170 min, where the storage modulus remained particularly low.

Consistent with the steady shear measurements, THF-based dilutions exhibited higher storage moduli and lower phase angles than acetone-based systems, indicating a stronger viscoelastic character during curing. For acetone-based dilutions at 4 g/ml, the storage modulus increased from 7.61 Pa at 160 min to 62.85 Pa at 190 min, while the phase angle decreased from 85.4° to 78.8°. In THF-based systems, the storage modulus increased from 12.04 Pa at 180 min to 128.7 Pa at 210 min, while the phase angle decreased from 85.5° to 71.0°.

#### Surface tension

**Fig. 4:**
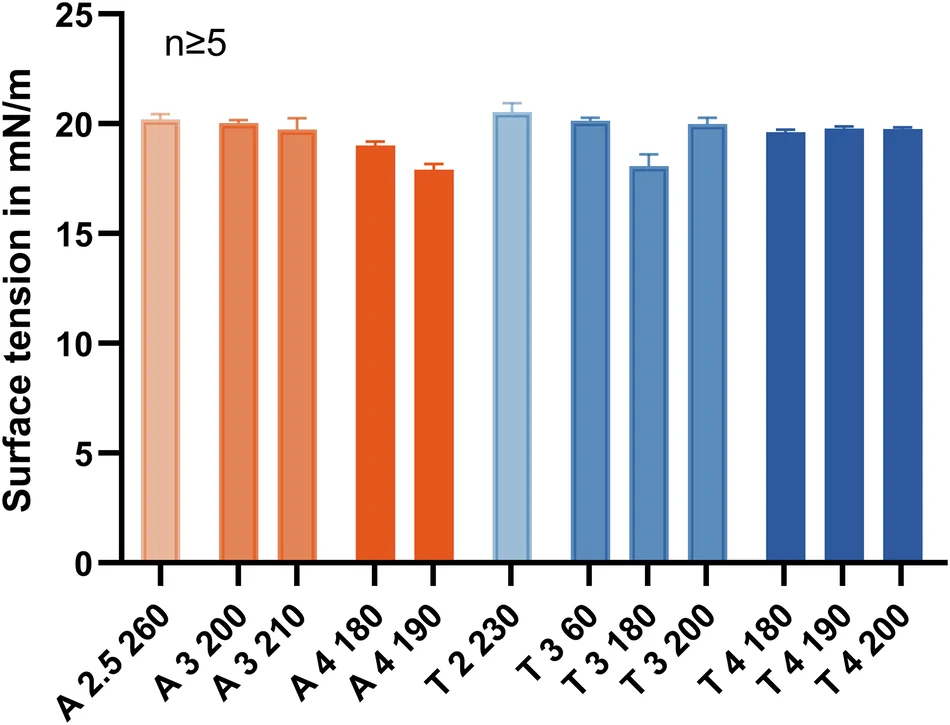
Temporal evolution of surface tension in silicone elastomer dilutions with acetone (orange/A) and THF (blue/T). Indices are given in this order: the diluent, the concentration in g/ml, and the curing time in min

Figure 4 summarizes the surface tension measurements for all conditions investigated. The sample labels indicate the diluent (A/T), polymer concentration (g/ml), and curing time (min). Surface tension remained nearly constant across all curing states investigated and ranged between 18 and 21 mN/m. A slight decrease in surface tension was observed with increasing polymer concentration, while THF-based dilutions exhibited marginally higher values than acetone-based systems. Overall, no systematic influence of curing time on surface tension was observed.

### Electrospinning process stability and morphology

The following section presents the electrospinning behavior of silicone elastomer dilutions with a concentration of 4 g/ml in detail. Results for all additional concentrations are summarized in Table 1.

#### Process evolution during electrospinning

Process stability during electrospinning was evaluated by visual observation. For acetone-based dilutions, a Taylor cone formed at a curing time of 160 min. However, no stable polymer jet could be established. At curing times of 170 and 180 min, a continuous polymer jet formed but repeatedly broke during processing. Electrospinning experiments at 190 min were not possible because the material lost flowability at the beginning of the process. In contrast, THF-based dilutions produced stable and continuous polymer jets at all investigated curing times. Acetone-based systems exhibited bending instabilities characteristic of conventional electrospinning, whereas THF-based systems showed markedly reduced or completely absent bending instabilities. Furthermore, the lateral jet motion decreased with increasing curing time for THF-based dilutions.

#### Fiber morphology

**Fig. 5:**
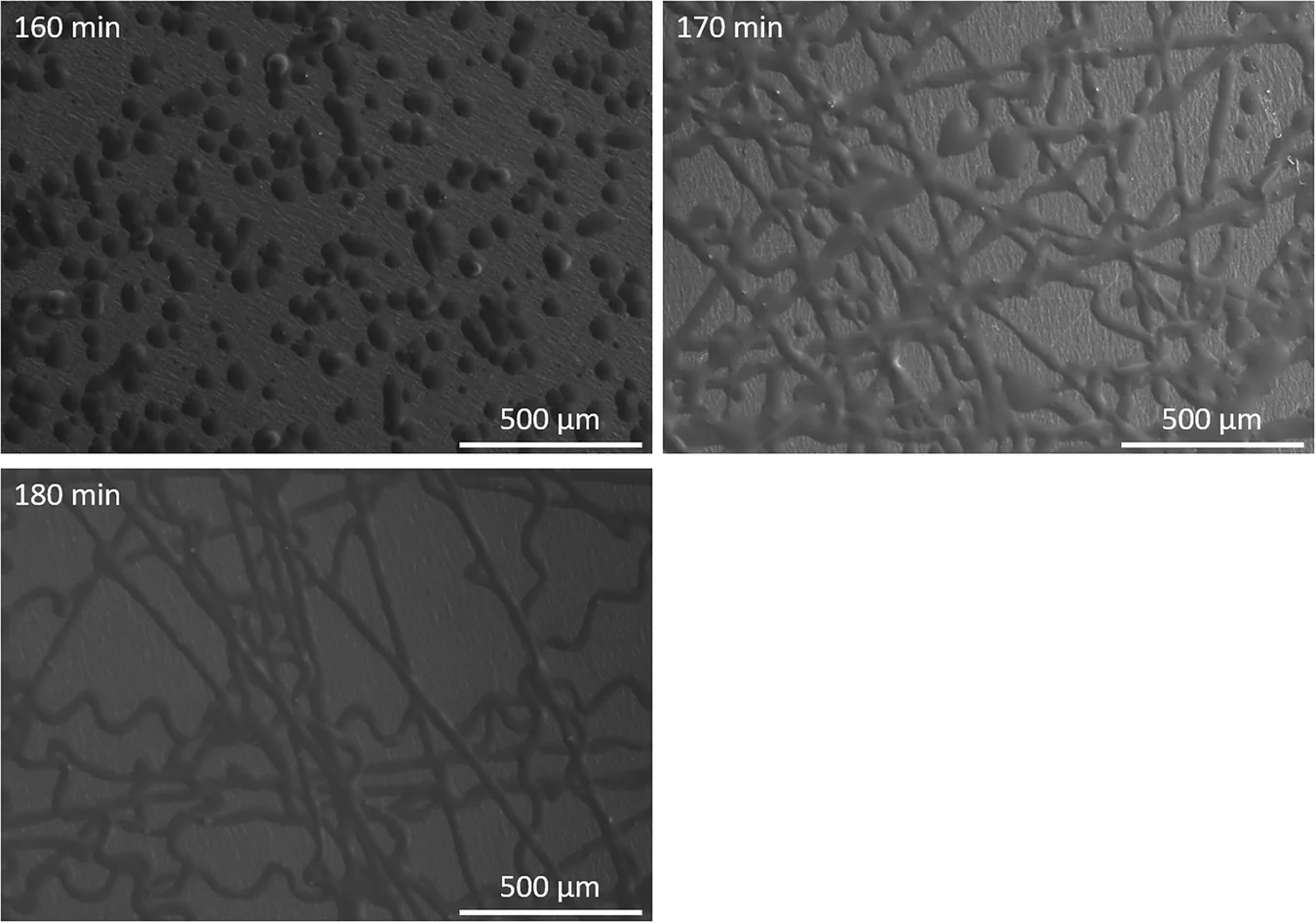
SEM images from electrospinning experiments with silicone elastomer/acetone dilutions at a concentration of 4 g/ml for curing times of 160, 170 and 180 min. A transition from electrospraying (160 min) to electrospinning (180 min) was observed. The 170 min experiment presents a transition zone with short, fragmented fibers. The fibers typically exhibit a ribbon-like structure due to partial sagging prior to complete curing. The magnification is 70x

**Fig. 6:**
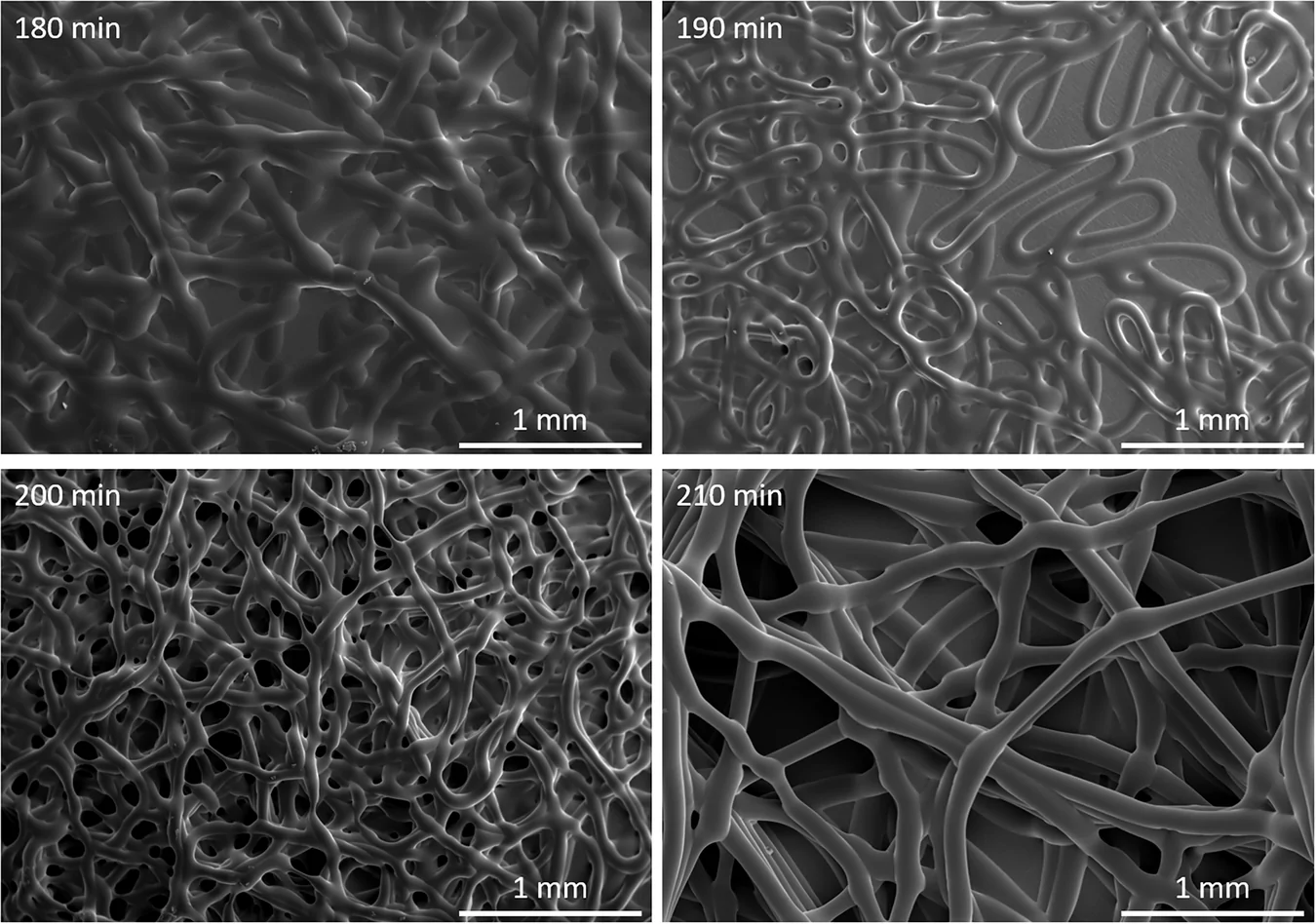
SEM images from electrospinning experiments with silicone elastomer/THF dilutions at a concentration of 4 g/ml for curing times of 180, 190, 200 and 210 min. At a curing time of 180 min, a transition region characterized by fragmented fibers and significant sagging was observed. Electrospinning commences at a curing time of 190 min. For curing times of 200 min or longer, a well-defined fiber morphology with minimal sagging and connected fiber interspaces is evident. The magnification is 35x

SEM images of electrospun fibers produced from 4 g/ml dilutions are shown in Figs. 5 and 6 for acetone- and THF-based systems, respectively. Processing regimes and fiber diameters for all additional concentrations are summarized in Table 1.

For acetone-based systems, curing time strongly influenced both the processing regime and resulting fiber morphology. At 160 min, the process corresponded to electrospraying, characterized by isolated beads. At 170 min, a transition regime between electrospraying and electrospinning was observed, consisting of droplets, short fiber fragments, and a limited number of continuous fibers. Some fragmented fibers exhibited a twisted, corkscrew-like morphology, which may be attributed to elastic recoil. At 180 min, continuous electrospinning was achieved, producing predominantly homogeneous fibers, although distorted structures were still present. Across all acetone-based experiments, the fibers exhibited ribbon-like morphologies accompanied by pronounced sagging. In multilayer deposits, the upper fiber layers increasingly collapsed, leading to poor structural integrity and poorly interconnected pore structures.

THF-based systems exhibited electrospinning behavior at all investigated curing times. However, the resulting fiber morphology evolved substantially during curing. At 180 min, fiber-like structures formed but displayed severe sagging and poorly interconnected pore structures. At curing times of 200 and 210 min, uniform round fibers with minimal sagging and well-defined interconnected pores were obtained. Fibers produced from THF-based dilutions exhibited substantially larger diameters than those produced from acetone-based systems. In addition, fiber diameter increased considerably with curing time.

#### Correlation between rheology and electrospinning behavior

**Fig. 7:**
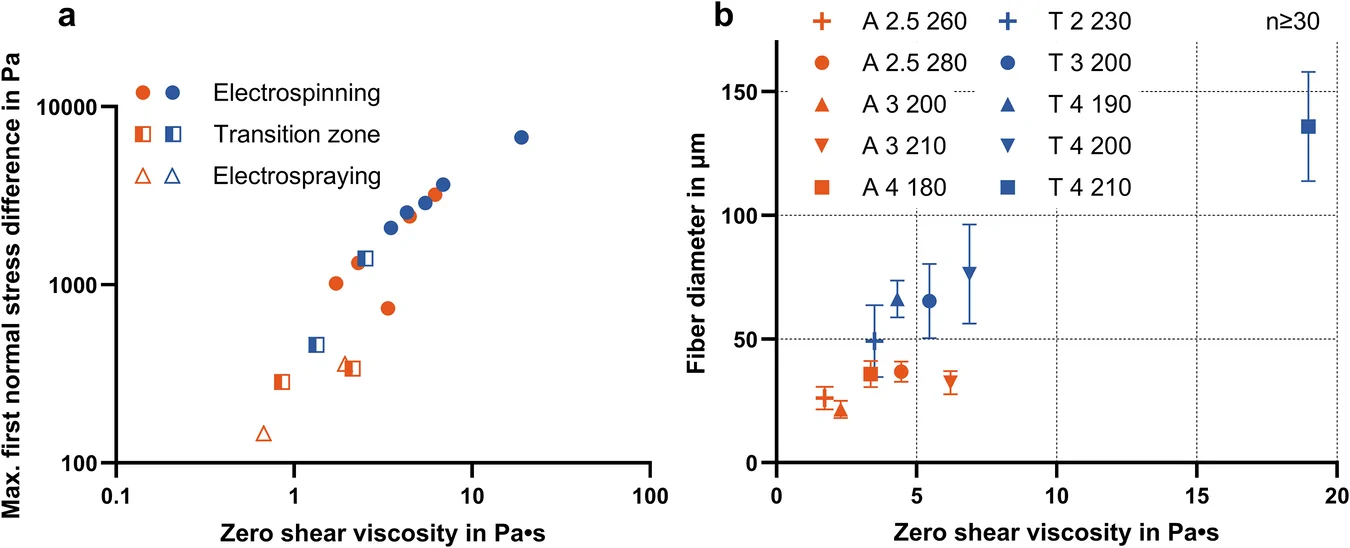
**a** Process regime of all conducted electrospinning experiments as a function of zero-shear viscosity and maximum first normal stress difference for dilutions in acetone (orange) and THF (blue). **b** Median fiber diameter with standard deviation for all electrospinning experiments with stable fiber morphology in relation to zero-shear viscosity of the dilution

Comparison of the rheological and electrospinning results revealed a strong relationship between viscoelastic material properties, process stability, and resulting fiber morphology. As shown in Fig. 7a, the processing regime of all experiments can be represented as a function of viscoelastic parameters (here zero-shear viscosity and maximum first normal stress difference). With the exception of acetone dilutions at 2 g/ml, a stable electrospinning window could be identified for all investigated dilutions. Stable electrospinning emerged for dilutions above 1.27 Pa·s (shear viscosity) and 1016 Pa (N_1_) for acetone as well as for dilutions above 3.5 Pa·s and 2083 Pa for THF. THF-based dilutions substantially expanded the processing window by increasing the time interval between the onset of stable electrospinning and loss of flowability. The improved electrospinning stability observed for THF-based systems coincided with higher viscosity, larger first normal stress differences, higher storage moduli, and lower phase angles compared to acetone-based systems.

Figure 7b summarizes the measured fiber diameters for all experiments exhibiting electrospinning behavior. Fiber diameter was highly sensitive to the choice of diluent. THF-based systems produced fibers with diameters ranging from 49 to 136 µm, whereas acetone-based systems yielded significantly smaller fibers between 22 and 37 µm. Even at comparable zero-shear viscosity, THF-based systems produced substantially larger fibers. For example, samples A 3 210 and T 3 200 exhibited similar viscosity of 6.21 and 5.46 Pa·s, respectively, but yielded median fiber diameters of 32.39 and 65.29 µm.

## Discussion

### Evolution of viscoelastic behavior during curing

The rotational rheological measurements demonstrated a continuous increase in viscosity and shear thinning with increasing curing time. Silicone elastomer dilutions immediately after preparation exhibited nearly Newtonian flow behavior, consistent with previous reports for Sylgard 184 prepolymer systems [23, 24]. The increase in viscosity observed at low shear rates is primarily attributed to operation near the rheometer torque limit, whereas the viscosity plateau at higher shear rates reflects the intrinsic flow behavior of the uncured prepolymer. Sylgard elastomer base consists of linear, vinyl-terminated PDMS prepolymers [25]. The nearly Newtonian behavior prior to curing suggests that the molecular weight and chain architecture of the prepolymer are insufficient to generate substantial intermolecular entanglement. During curing, however, hydrosilylation progressively transforms the system into a partially crosslinked and increasingly branched elastomer precursor [26]. The resulting increase in viscosity and shear thinning may therefore be attributed to both increasing intermolecular entanglement and the gradual formation of a transient elastomeric network. Oscillatory rheological measurements support this interpretation. All investigated systems remained predominantly viscous over most of the curing process, as indicated by phase angles close to 90°. Only as the gel point was approached did the storage modulus increase substantially, while the phase angle decreased, indicating the emergence of elastic contributions. Similar behavior has previously been reported for curing silicone elastomers, where rheological changes become pronounced primarily in the vicinity of gelation despite ongoing crosslinking at earlier stages [25, 27]. The sharp increase in viscoelastic markers immediately prior to gelation observed here is therefore consistent with described curing kinetics of PDMS elastomers. Importantly, the rapid rheological evolution near the gel point implies that electrospinning reproducibility is highly sensitive to curing time. Small temporal deviations within the stable processing window may therefore result in substantial changes in viscoelastic behavior and consequently in electrospinning stability and fiber morphology.

The first normal stress difference provides additional insight into the evolution of nonlinear elastic behavior during curing. In uncured silicone elastomer dilutions, no measurable first normal stress differences were detected, indicating the absence of significant elastic stresses. With ongoing curing, however, a pronounced threshold behavior emerged, where the first normal stress difference remained negligible over a broad shear-rate range before increasing sharply beyond a critical shear rate. In contrast to conventional polymer solutions, where the first normal stress difference typically increases continuously with shear rate to an approximately quadratic scaling [28], the present system exhibited measurable nonlinear elasticity only once a sufficient degree of transient network connectivity had developed. As curing progressed, the onset shear rate for measurable elasticity shifted towards lower values, indicating that elastic stresses could increasingly be generated at lower deformation rates. This evolution of nonlinear elasticity coincided with the experimentally observed transition from electrospraying to electrospinning. The results therefore suggest that stable electrospinning in partially cured PDMS systems is governed more strongly by the emergence of elastic restoring forces than by viscosity alone. During electrospinning, sufficient elasticity is required to stabilize the jet against capillary breakup and prevent fragmentation into droplets or short fiber segments [29, 30].

The distinction between oscillatory and rotational rheology is therefore particularly important. While the storage modulus obtained from small-amplitude oscillatory measurements characterizes linear viscoelastic behavior, the first normal stress difference reflects nonlinear elastic effects under large deformation. Since electrospinning involves strong jet stretching and extensional deformation, nonlinear elastic properties are likely more relevant for jet stabilization than linear viscoelastic parameters alone [31]. Although the actual deformation rates within the electrospinning process cannot readily be quantified and the flow downstream of the Taylor cone is predominantly extensional rather than shear-dominated, the observed correlation between measurable first normal stress differences and stable electrospinning strongly supports the importance of nonlinear elasticity for fiber formation in electrospinning of silicone elastomers.

### Role of surface tension

Surface tension is generally considered an important parameter in electrospinning because it influences Taylor cone formation, jet initiation, and capillary breakup [32]. High surface tension can promote bead formation and jet instability, whereas low surface tension generally favors continuous fiber formation. However, the present results indicate that surface tension played only a minor role in the observed processing differences. All investigated solutions exhibited comparatively low surface tensions between 18 and 21 mN/m, with only minimal variation as a function of polymer concentration or curing time. The slightly higher surface tension observed for THF-based systems can be attributed directly to the intrinsic surface tension of the diluent itself. Since substantial differences in electrospinning behavior occurred despite nearly constant surface tension, the transition from electrospraying to stable electrospinning is more plausibly explained by the evolution of viscoelastic properties than by changes in surface tension.

### Influence of diluent on rheology and electrospinning behavior

The comparison between acetone- and THF-based systems demonstrates that the choice of the diluent strongly influences both rheological behavior and electrospinning stability. THF-based dilutions consistently exhibited higher viscosity, larger first normal stress differences, higher storage moduli, and lower phase angles within the investigated curing range. Swelling studies reported in the literature identify THF as a substantially better solvent for PDMS than acetone [33]. Enhanced polymer–diluent interactions in THF are expected to produce more expanded polymer coil conformations, thereby increasing intermolecular overlap and entanglement density. The stronger viscoelastic response observed in THF-based systems therefore suggests that viscoelasticity in partially cured PDMS systems is not governed exclusively by chemical network formation, but is also strongly influenced by solvent-dependent chain conformations and intermolecular interactions. This interpretation is consistent with the electrospinning experiments. THF-based systems exhibited broader stable processing windows and enabled fiber formation even at concentrations of 2 g/ml, whereas acetone-based systems failed to produce fibers at the same concentration. Furthermore, THF-based systems generated more stable polymer jets and more homogeneous fiber morphologies.

The substantial differences in fiber diameter observed between THF- and acetone-based systems indicate that diluent conductivity likely contributed to the fiber diameters. Even within overlapping viscosity ranges, THF-based systems produced significantly larger fiber diameters. Since Sylgard 184 contains almost no intrinsic charge carriers, the higher electrical conductivity of acetone compared with THF is expected to increase the charge density carried by the electrospinning jet, thereby promoting greater jet stretching and smaller fiber diameters, as displayed in Fig. 7b. This interpretation is consistent with reported electrical conductivities of the pure solvents [17] and the established relationship between solution conductivity and fiber diameter in electrospinning [34].

### Jet stability and fiber morphology

Substantial differences were observed between electrospinning of partially cured PDMS elastomers and conventional electrospinning of thermoplastic polymer solutions. The reduced bending instabilities observed for PDMS systems were likely governed by a combination of low jet charge density and high elastic restoring forces. Acetone-based systems exhibited pronounced bending instabilities and produced substantially smaller fiber diameters, indicating stronger electrostatic jet stretching than in THF-based systems. In contrast, THF-based systems exhibited markedly reduced or entirely absent visible bending instabilities. The progressive suppression of bending instabilities with increasing curing time in THF-based systems further suggests that elastic restoring forces counteracted jet thinning during flight. Similar effects have previously been reported for polymer systems with increasing polymer concentration (and thus, elasticity), where the onset of bending instabilities is delayed [35]. Interestingly, the suppression of bending instabilities in solvent-based electrospinning of silicone elastomers may enable more predictable fiber deposition behavior. Consequently, this process may offer conceptual parallels to additive manufacturing approaches such as melt electrowriting.

Distinct corkscrew-like fiber morphologies were observed in acetone-based systems, in which repeated jet breakage also occurred during electrospinning. These structures likely originate from elastic recoil of the polymer jet following fragmentation. After jet breakage, elastic contraction of the partially cured jet may occur before complete solidification on the collector, resulting in the observed helical morphologies. In THF-based systems, continuous jets were maintained throughout processing, and neither jet breakage nor corkscrew-like fibers were observed. The ribbon-like morphology and pronounced sagging observed predominantly in acetone-based systems further indicate insufficient structural stabilization during deposition. In contrast, THF-based systems produced increasingly round and structurally stable fibers with ongoing curing, suggesting that enhanced viscoelastic stabilization suppresses collapse of the deposited fibers prior to complete solidification.

Although the present process enabled the fabrication of structurally defined silicone elastomer nonwovens, the resulting fiber diameters (22-136 µm) are comparatively large relative to those typically reported for electrospun thermoplastic polymers [36, 37]. For cochlear implant electrode coatings, large fiber diameters may influence both the overall coating thickness and the cellular response to the surface microstructure. Consequently, optimization of fiber diameter may become important for balancing structural integrity with implant geometry and biological performance. The present study focused on establishing the relationship between curing-induced rheological evolution and electrospinnability. Future in vitro investigations will therefore assess how fiber diameter influences fibroblast behavior and determine the diameter range that provides the most favorable biological response while remaining compatible with cochlear implant electrode geometries.

Overall, the results demonstrate that electrospinning of partially cured PDMS systems is governed primarily by the evolution of the viscoelastic behavior during curing. The transition from electrospraying to stable electrospinning coincided with the emergence of measurable elastic stresses, indicating that elastic restoring forces are essential for maintaining jet integrity. In addition, the choice of diluent influenced the accessible electrospinning window through its effects on polymer conformation, intermolecular interactions, electrical properties, and viscoelastic response. The present study demonstrates that the challenge of electrospinning pure addition-curing silicone elastomers can be addressed through precise control of the pre-curing state. Rather than representing a limitation, the time-dependent evolution of rheological properties defines a processability window in which stable jet formation and fiber shape retention can be achieved simultaneously. This approach enables the fabrication of porous silicone nonwovens with preserved fiber morphology and may facilitate the development of implant surface coatings designed to modulate the tissue response while maintaining electrical functionality.

## Conclusion

The findings of this study demonstrate that precise modulation of the rheological properties through the selection of the pre-curing time for electrospinning of silicone elastomers facilitates the fabrication of nonwovens with sufficient fiber shape retention. As the gel point is approached, silicone elastomer dilutions demonstrate increased viscosity and pronounced elastic properties. On one hand, this stabilizes the formation and maintenance of a polymer jet from the Taylor cone, leading to a transition from electrospraying to electrospinning. Furthermore, this process reduces the remaining reaction path of PDMS elastomer systems towards the gel point for the in situ curing mechanism. Faster in situ curing results in the counteraction of fiber sagging and the production of PDMS nonwovens with defined fiber morphology and interconnectivity of pores. The process described herein offers significant potential for the production of hydrophobic surface coatings in the medical field, as comparable platinum-catalyzed, addition-curing silicone elastomers are available in medical-grade quality. The fiber diameter in PDMS elastomer electrospinning can be varied in magnitudes by selecting the polarity of the diluent. This opens the field to various applications and morphologies for this process. The polymer concentration exhibits negligible influence on the diameter compared to the choice of the diluent. For electrospinning in high polarity diluents, further improvement in the in situ curing strategy might be necessary to avoid sagging and ribbon-like fiber morphologies. Future research will focus on investigating how fibroblasts interact with the electrospun surface modifications. Additionally, the flow below the Taylor cone during electrospinning is dominated by elongational flows. Further elongational rheological data would be valuable for characterizing the silicone elastomer dilutions and understanding their behavior within the fiber jet.

## Supplementary information

Supplementary Information

## Data Availability

The raw data from the rheological tests will be provided upon request.
