# Supplementary material for: Curing-state-dependent rheology governs electrospinning of silicone elastomers for hydrophobic surface coatings
Source: J Mater Sci Mater Med. 2026 Nov 5;37(1):136. doi: 10.1007/s10856-026-07125-z (PMC13740063; doi:10.1007/s10856-026-07125-z)
Supplement: Supplementary file 1 [file 10856_2026_7125_MOESM1_ESM.pdf]

Curing-state-dependent rheology governs electrospinning of silicone elastomers for hydrophobic surface coatings

Journal of Materials Science: Materials in Medicine

Supplementary information

Jan F. Drexler[1]*, Yan Zhang[1], Florian Rummel[2], Gesine Hentschel[1], Tom Bode[1], Marc Mueller[1], Gerrit Paasche[3,4,5], Birgit Glasmacher[1]

[1]Institute for Multiphase Processes, Leibniz University Hannover, An der Universität 1, Garbsen, 30823, Germany.

[2]NETZSCH-Gerätebau GmbH, Wittelsbacherstraße 42, Selb, 95100, Germany.

[3]Department of Otorhinolaryngology, Head and Neck Surgery, Hannover Medical School, Carl-Neuberg-Straße 1, Hannover, 30625, Germany.

[4]Lower Saxony Center for Biomedical Engineering, Implant Research and Development, Stadtfelddamm 34, Hannover, 30625, Germany.

[5]Hearing4all, Hannover Medical School, Carl-Neuberg-Straße 1, Hannover, 30625, Germany.

Corresponding author: Jan F. Drexler drexler@imp.uni-hannover.de

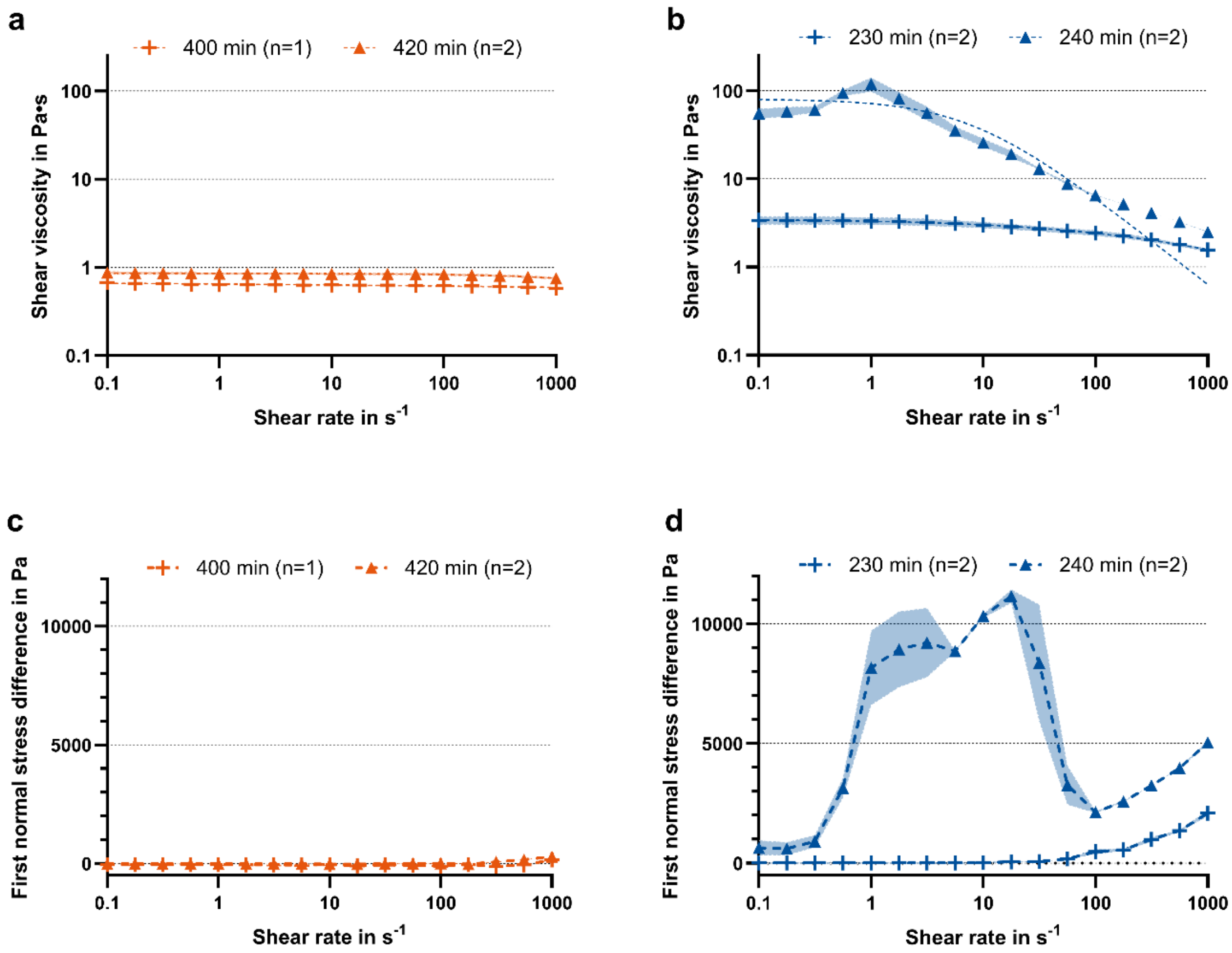

Figure S1: Temporal evolution of shear viscosity and first normal stress difference of silicone elastomer dilutions with a concentration of 2 g/ml in acetone (orange, a and c) and THF (blue, b and d). For viscosity measurements, the dashed lines indicate the fit according to the Cross model. If several measurements could be performed with the same dilution, the data points indicate the median values and the envelope curves indicate the total range.

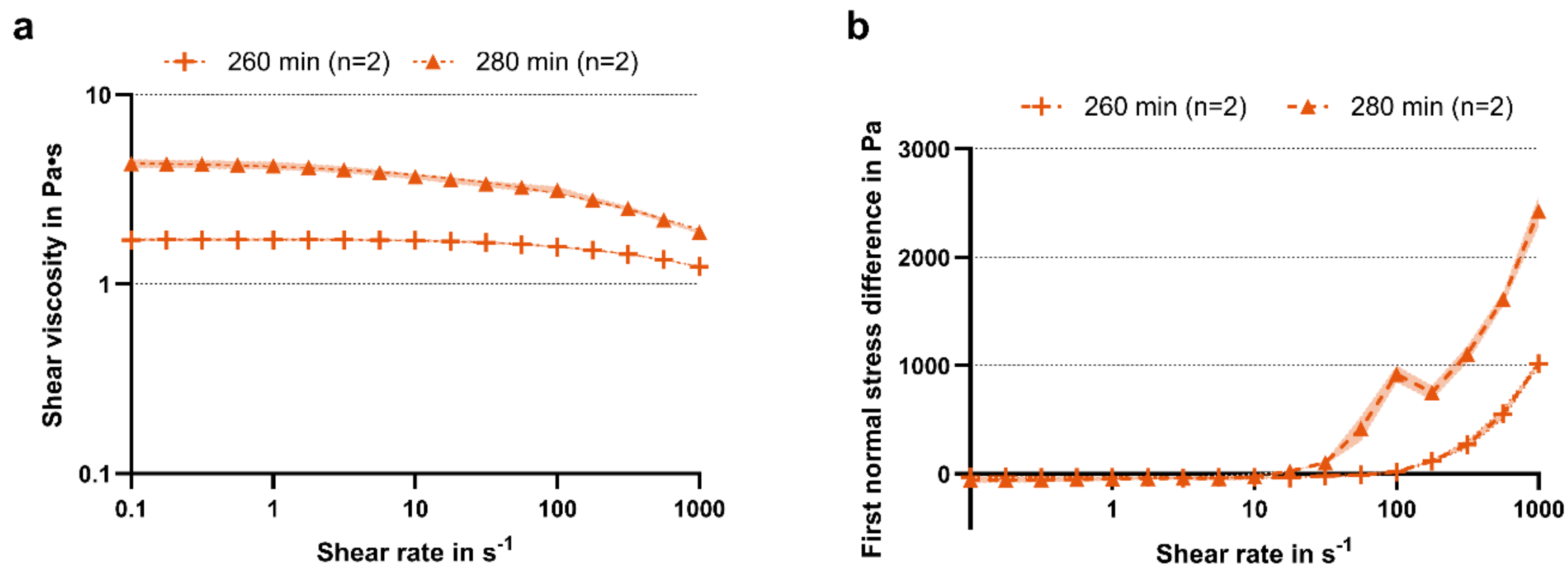

Figure S2: Temporal evolution of shear viscosity (a) and first normal stress difference (b) of silicone elastomer dilutions with a concentration of 2.5 g/ml in acetone. For viscosity measurements, the dashed lines indicate the fit according to the Cross model. If several measurements could be performed with the same dilution, the data points indicate the median values and the envelope curves indicate the total range.

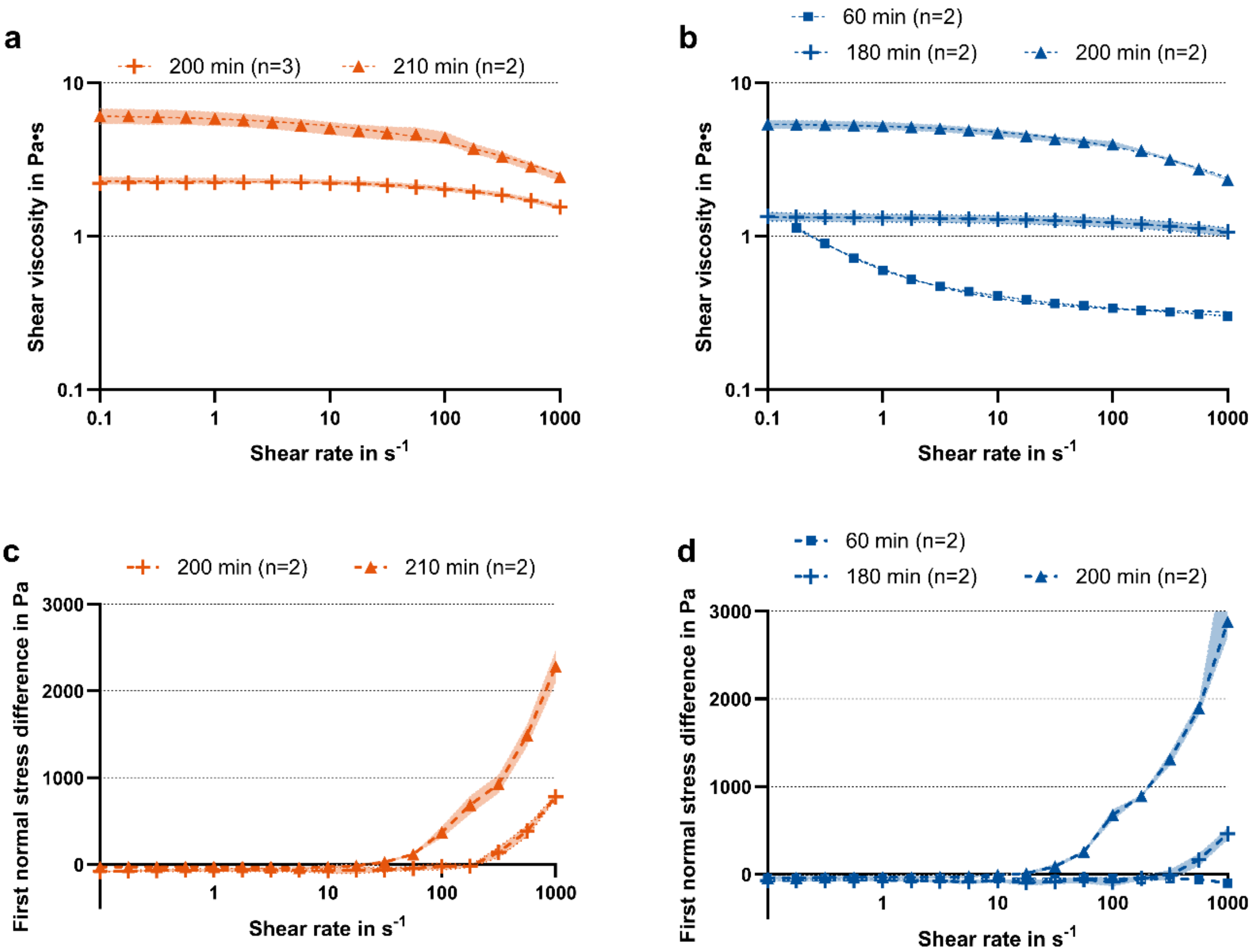

Figure S3: Temporal evolution of shear viscosity and first normal stress difference of silicone elastomer dilutions with a concentration of 3 g/ml in acetone (orange, a and c) and THF (blue, b and d). For viscosity measurements, the dashed lines indicate the fit according to the Cross model. If several measurements could be performed with the same dilution, the data points indicate the median values and the envelope curves indicate the total range.

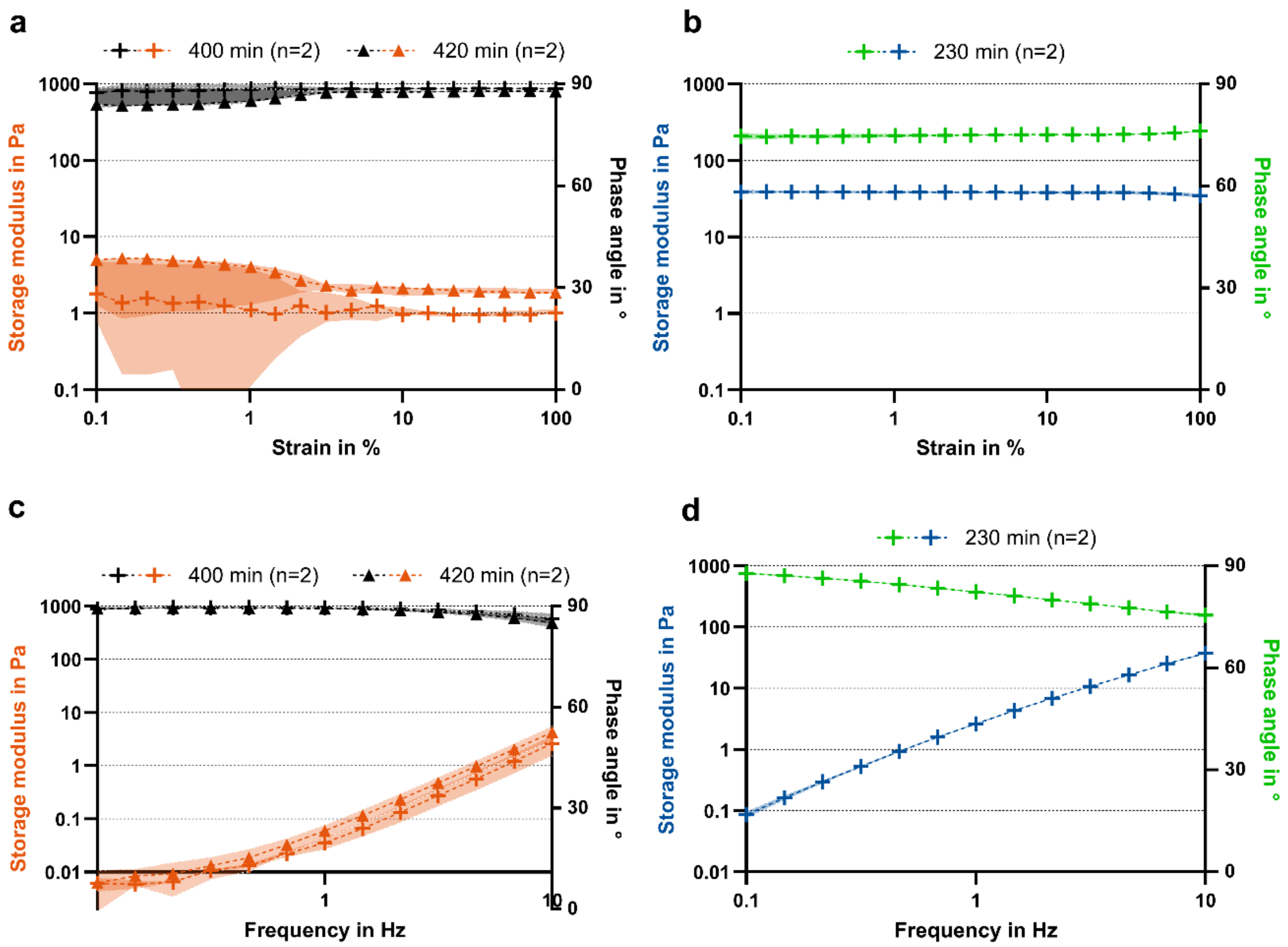

Figure S4: Left: Temporal evolution of storage modulus (orange) and phase angle (black) of silicone elastomer dilutions with a concentration of 2 g/ml in acetone in dependence of amplitude (a) and frequency (c). Right: Storage modulus (blue) and phase angle (green) of silicone elastomer dilutions with a concentration of 2 g/ml in THF in dependence of amplitude (b) and frequency (d). If several measurements could be performed with the same dilution, the data points indicate the median values and the envelope curves indicate the total range.

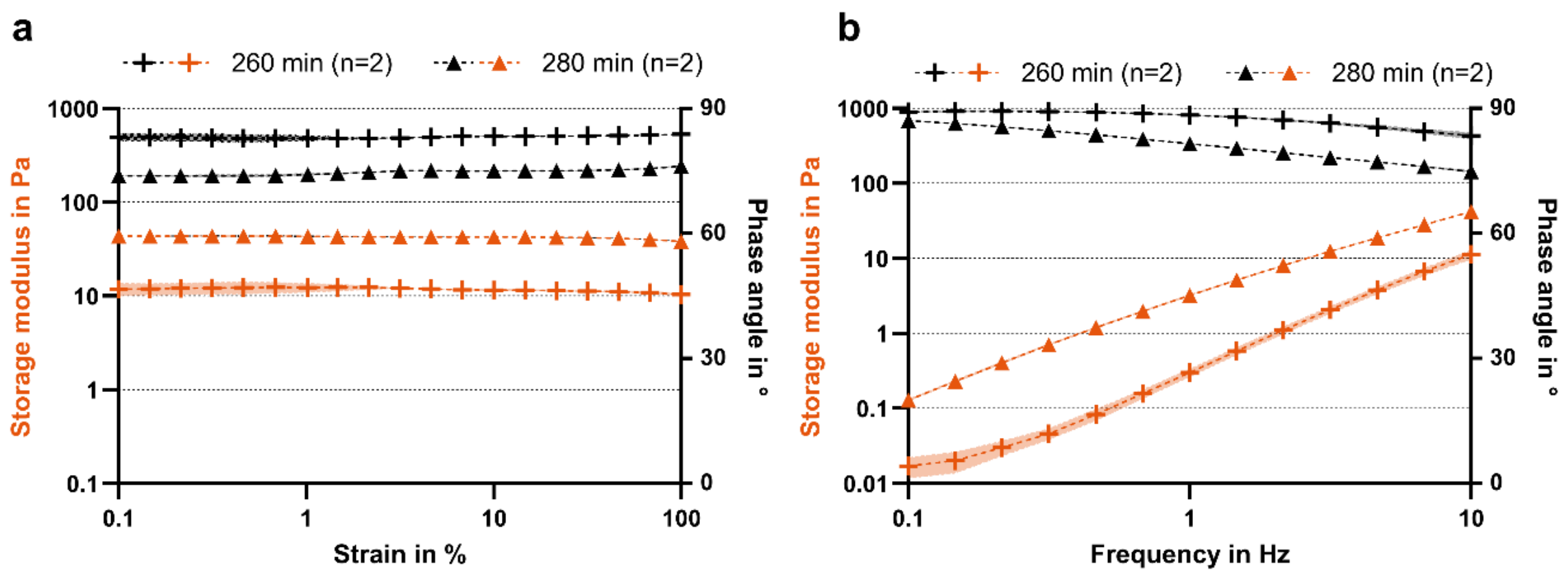

Figure 5: Temporal evolution of storage modulus (orange) and phase angle (black) of silicone elastomer dilutions with a concentration of 2.5 g/ml in acetone in dependence of amplitude (a) and frequency (b). If several measurements could be performed with the same dilution, the data points indicate the median values and the envelope curves indicate the total range.

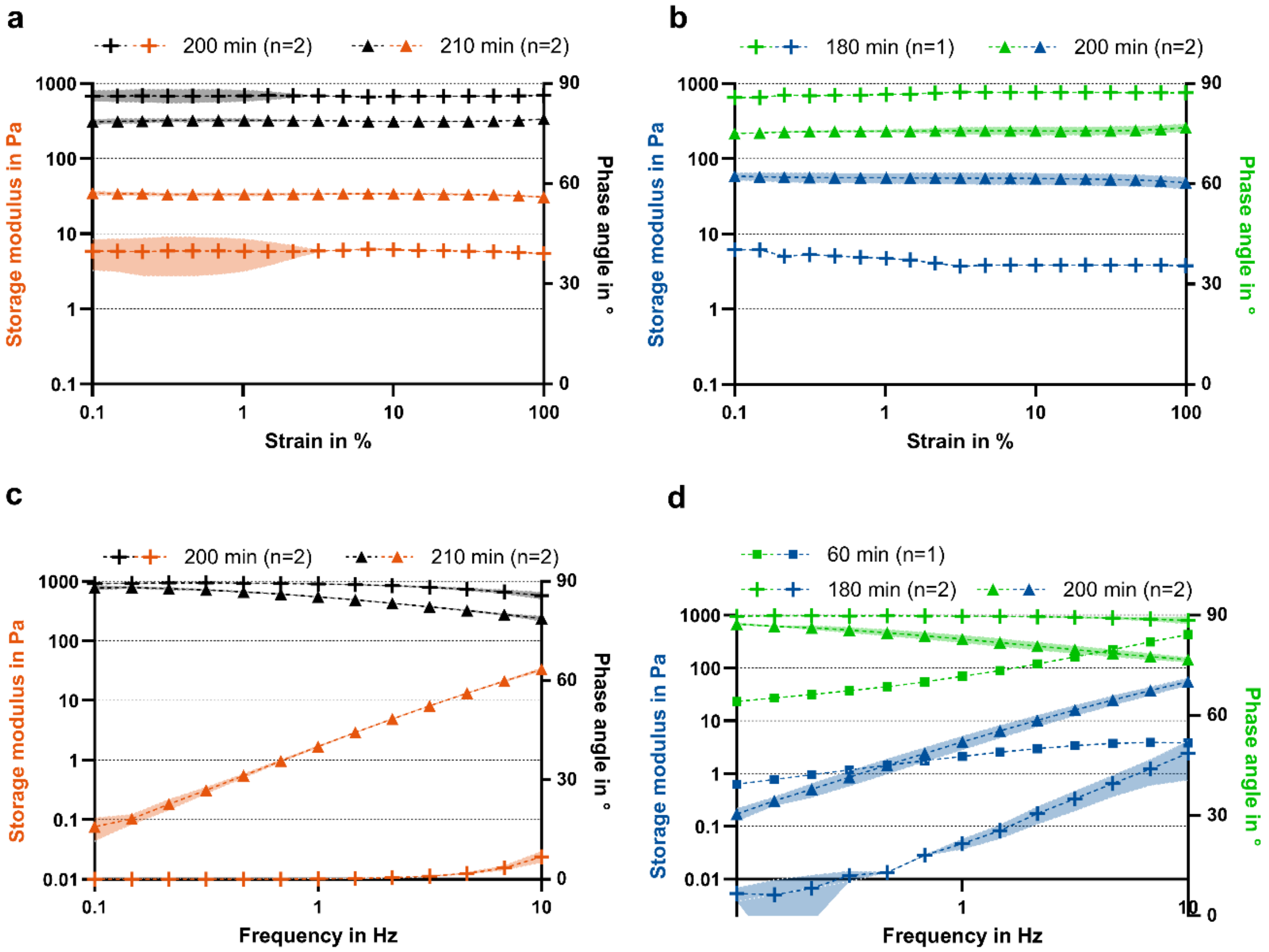

Figure S6: Left: Temporal evolution of storage modulus (orange) and phase angle (black) of silicone elastomer dilutions with a concentration of 3 g/ml in acetone in dependence of amplitude (a) and frequency (c). Right: Storage modulus (blue) and phase angle (green) of silicone elastomer dilutions with a concentration of 3 g/ml in THF in dependence of amplitude (b) and frequency (d). If several measurements could be performed with the same dilution, the data points indicate the median values and the envelope curves indicate the total range.
